# Targeting DNA Repair Pathways in Hematological Malignancies

**DOI:** 10.3390/ijms21197365

**Published:** 2020-10-06

**Authors:** Jehad F. Alhmoud, Ayman G. Mustafa, Mohammed Imad Malki

**Affiliations:** 1 Department of Medical Laboratory Sciences, Faculty of Applied Medical Sciences, Al-Ahliyya Amman University, Amman 19328, Jordan; j.alhmoud@ammanu.edu.jo; 2 College of Medicine, QU Health, Qatar University, P. O. Box 2713 Doha, Qatar; amustafa@qu.edu.qa

**Keywords:** DNA repair, DNA damage, hematological malignancies, apoptosis, mutation

## Abstract

DNA repair plays an essential role in protecting cells that are repeatedly exposed to endogenous or exogenous insults that can induce varying degrees of DNA damage. Any defect in DNA repair mechanisms results in multiple genomic changes that ultimately may result in mutation, tumor growth, and/or cell apoptosis. Furthermore, impaired repair mechanisms can also lead to genomic instability, which can initiate tumorigenesis and development of hematological malignancy. This review discusses recent findings and highlights the importance of DNA repair components and the impact of their aberrations on hematological malignancies.

## 1. Introduction

DNA damage leads to changes in the DNA double-helical structure [1]. Under normal physiological conditions, the DNA repair mechanism is activated in response to DNA damage in an attempt to correct the defected DNA and restore normal cell function [2]. This is crucial to cell cycle as it ensures that any genetic mutations are corrected before mitosis and not passed onto daughter cells [3]. Hematological malignancies (HM) account for approximately 10% of all newly diagnosed cancers and are usually characterized by genetic defect in the form of chromosomal translocation or breakpoint/fusion [4]. Many HM run a chronic relapsing course, culminating in therapy resistance with multiple lines of therapy [5]. Development of resistance to DNA-damaging chemotherapy agents such as cisplatin, cyclophosphamide, chlorambucil, and temozolomide can be somewhat mitigated with concurrent inhibition of DNA repair pathways, increasing cytotoxicity [6] and hence therapeutic efficacy [7]. The field of hematology-oncology is considered to be at the forefront of utilizing genomic tools to aid diagnosis, stratification of patients into treatment groups, and infer prognosis. Genetic testing is an integral part in classification of diseases with various techniques employed in the development of diagnostic workflows [8].

Genomic instability is one of the main drivers of hematological malignancy and is associated with inherited and acquired leukemias. The maintenance of genomic stability depends on the amount of continuous exposure to DNA-damaging agents and integrity of the immune system. Eighty percent of patients with chronic lymphocytic leukemia (CLL), a type of HM, display chromosomal anomalies, whereas this is seen in 50% of patients with acute myeloid leukemia (AML) or myelodysplastic syndrome (MDS) [9,10]. CLL along with AML/MDS exhibit genomic instability. It is also important to note that lymphoma is associated with hereditary diseases such as ataxia-telangiectasia (A-T), Nijmegen breakage syndrome (NBS), and bloom syndrome, which also exhibit genomic instability. Particular mutations such as ataxia telangiectasia mutated (ATM) gene are associated with the defects in DNA repair mechanisms and are present in various cancers including acute leukemia [11]. Several hematological cancers such as CLL and AML have a poor prognosis and therefore the use of PARP1 inhibitor, an inhibitor of the DNA repair pathway, has shown promising results in chemosensitization when treating with cytotoxic agents [12,13]. In this review, we highlight the most important components of the DNA repair pathway and the benefits of targeting them in improving the efficacy of anti-cancer therapy in hematological malignancies. Furthermore, we have discussed and summarized the use of new and emerging therapeutic agents in this field. 

## 2. Source of DNA Damage

The agents causing DNA damage can be endogenous or exogenous [14]. Reactive oxygen species (ROS) or ionizing radiation are some examples of DNA damaging agents [15]. Persistent exposure to genotoxic agents may cause disruption of covalent bonds between nucleotide sequences [16]. Resultant changes in the nucleotide sequences may lead to changes in genome replication, transcription, and hence aberrant expression of dysfunctional proteins, causing carcinogenesis [17,18]. 

Ionizing irradiation (IR) is one of the familiar sources of DNA damage, particularly during radiotherapy. IR leads to increased nuclear instability which causes direct and indirect DNA damage [19]. Several types of IR exist, such as alpha or beta particles and gamma radiation [20]. The energy released from the irradiation has the ability to damage proteins and nucleic acids resulting in double strand breaks (DSB) at the phosphodiester backbone of DNA [21,22]. The irradiation dose determines the level and complexity of DNA damage and the type of damage is influenced by the cancer therapy used [23].

DNA damaging agents are broadly categorized into two categories: clastogens and aneugens. Clastogens are mutagenic agent stimulating disruption or breakages of chromosomes, while aneugens are substances that cause a daughter cell to have an abnormal number of chromosomes. Chromosomal breaks are caused by clastogens and result in acentric chromosomal fragments, while aneugens causes aneuploidy, which impacts cell division and the mitotic spindle apparatus [24].

## 3. The DNA Damage Response (DDR)

Thousands of single strand breaks (SSBs) and approximately 10–50 DSBs occur in each cell daily, requiring correction by cellular mechanism of DDR [25]. Therefore, DDR plays a crucial role in maintaining genomic integrity. DDR comprises a group of pathways involved in detecting DNA damage, identifying their location, and promoting their effect. Disruption of DDR can lead to disease pathogenesis, including immune dysfunction, neurological deterioration, progeria, and predisposition to cancer [26]. Cell survival or replication is affected by genetic alterations, particularly when it occurs in oncogenes, tumor-suppressor genes, and genes that regulate cell cycle [27]. The sites of DNA damage are identified by DNA repair proteins that are part of the cell cycle checkpoints leading to activation of repair or apoptosis pathways [28].

Important proteins can be activated in response to DNA damage and are essential in controlling the rate of cell growth. These proteins are considered as the main components of DDR-signaling in mammalian cells, which include protein kinases ATM and ATR, which play a crucial role in repair of DNA damage [29]. These components are stimulated by DSBs and replication protein A (RPA) that attach to single-stranded DNA (ssDNA) [30]. ATM/ATR are responsible for targeting protein kinases called checkpoint kinase, CHK1 and CHK2T, as both functionally facilitate replication fork stabilization during DNA replication and repair. Inhibition of CDKs by cell cycle checkpoints leads to cell arrests or reduction of cell-cycle progression in G1-S, intra-S, and G2-M. DNA repair pathways are controlled by cell-cycle checkpoints before the cell enters the stage of mitosis. Furthermore, ATM/ATR cell signaling plays a role in the enhancement of transcription of DNA repair proteins and post-translational modification by phosphorylation, acetylation, or ubiquitination [31]. 

Proteomic studies have shown that DDR is involved in regulating various cellular processes in order to identify abnormal ATM/ATR-mediated phosphorylation sites [32]. The stimulation of oncogenes or dysfunction of tumor-suppressors genes are responsible for abnormal cell proliferation. ATR/ATM-mediated signaling activated following exposure to stress, initiates cell apoptosis, but also causes cell progression in vivo [33,34]. Within the context of cancer, DDR is stimulated at an early stage of tumor growth to prevent cancerous cells from proliferating [35]. However, inactivation of DDR pathways due to mutagenesis or epigenetic changes leads to proliferation of malignant cells, causing tumorigenesis. 

When the level of DNA damage exceeds the ability of the repairing mechanism to fix the damaged site, DDR signals stimulate cell apoptosis, leading to activation of checkpoints resulting in reduction of the activity of cyclin-dependent kinase (CDK) and therefore, cell death or cell-cycle arrest. Activation of p53 transcription is one of the possible anti-tumor response strategies [33,36]. Moreover, chromatin controls DDR and structural changes occur in response to DNA damage [37]. An example of the actions that occur on the site of DNA damage is the phosphorylation of serine-139 of the histone H2A variant H2AX by ATM/ATR/DNA-PK, on chromatin. The ubiquitin-adduct formation in the DNA damaged regions, the recruitment of DDR factors and chromatin-modifying components are responsible for the enhancement of DSB repair and improve DSB signaling [31]. Unusually, activation of ATM also leads to chromatin relaxation at sites of DSBs [38]. Furthermore, H2AX tyrosine-142 phosphorylation is an example of activated DDR [39]. 

Several metabolites induce DNA damage and impair genomic stability including ROS, nitrogen species, carbonyl species, and lipid peroxidation products, such as 4-hydroxynonenal. Genomic alteration can also occur under normal physiological condition. Somatic recombination through V(D)J recombination and somatic hyper-mutation are important processes which occur in B and T-cells. This results in production of diversity of immunoglobulins and T-cell receptors, required for antigen recognition or through DNA topoisomerase 2 in order to control the integrity and structure of DNA within the nucleus [40].

Aging has been linked to telomere shortening [41]. Epithelial cancer cells, which have a rapid mitotic rate, result in shedding and high turnover of the epithelium, particularly in elderly patients [42]. In early cancerous lesions, the stimulation of epithelial carcinogenesis is caused by critical shortening of telomeres or abnormality in telomere function, leading to induction of tetraploidization, which plays a role in chromosomal instability (CIN) and tumorigenesis [43,44,45,46]. In tetraploidy, there is a tendency to lose chromosomes gradually when the chromosomes are disturbed randomly through division to daughter cells, leading to CIN [47]. Several cancers display some form of CIN suggested to be caused by the shortening of telomeres and, as consequence, chromosomal fusions [48].

DSB exposes the genome, especially oncogenes, to various DNA-damaging metabolites causing a constant accumulation of CIN [49]. Genomic instability and dysfunction of DDR pathway in later stages of cancer development are associated with severe hypoxia [50]. When inherited, DDR abnormalities can contribute to the “mutator phenotype” of many cancers. Together, these evidences highlight that a deficient DDR system is associated with increase of defected DNA in human cancerous cells. Therefore, DDR is an essential anti-cancer component and a potential target for anti-cancer therapy [49]. 

## 4. DNA Damage Regulators and Cell Cycle Checkpoints

Cell-cycle checkpoints control the cell cycle in maintaining its order and progression, ultimately ensuring chromosomal integrity [51]. These checkpoints are present in three locations within the cell cycle including G1/S, S, and G2/M. The G1/S checkpoint prevents the replication of the damaged DNA, and the main factor that causes a G1/S arrest and apoptosis is the tumor-suppressor protein, p53 [52]. However, the S phase DNA damage checkpoint (SDDC) is initiated when the DNA damage occurs throughout the early part of the DNA S phase. Thus, the role of S phase checkpoint depends on controlling and withstanding damage at the time of DNA replication than repairing the damage [53]. The G2/M checkpoint is stimulated in order to repair the damaged DNA in S and G2 phase prior to cell division. G2 phase plays an essential role in protecting daughter cells from the transmission of impaired genetic material [54]. Several studies in mammalian cells revealed CHK1, CHK2 Mre11, Rad50, Rad51, Rad54, Ku family, and many others as checkpoint proteins in addition to p53, which acts as an essential checkpoint regulator. They all play an important role in repair of double strand breaks [55]. *TP53* gene regulates various growth arrest checkpoints such as G1/S and G2/M in addition to DNA-damage responsive genes such as p21, MDM2, and GADD45 [56]. 

Generally, the interaction between tumor-suppressor genes, repair proteins, and checkpoint regulators explain the reason behind the pleiotropic phenotypes of mutated cells [57]. Extrinsic and intrinsic factors cause programmed DNA damage during meiotic recombination or immunoglobulin gene rearrangements. Other examples of exogenous and endogenous DNA damaging agents that are responsible for causing DNA damage and activating cell cycle checkpoints include ultraviolet (UV) light, methyl-methane-sulfonate (MMS), cisplatin, neocarzinostatin (NCS), and ROS [58]. DNA repair mechanism is crucial for normal cell cycle progression as it limits and controls genomic aberration and cancer development. The DNA has to be repaired before the cell splits into daughter cells to avoid establishment of cells with mutated genetic material. 

## 5. DNA Repair

Defects in DNA repair mechanisms has been implicated in the pathogenesis of several hematological malignancies (HM) as well in therapy resistance [59,60]. In addition, poor prognosis in several cancers such as lymphoid malignancies is associated with irregular or changes in the epigenetic mechanism such as histone post-translational modifications, particularly histone H3 lysine-27 trimethylation (H3K27me3) [61,62]. Another finding showed that some lymphoid malignancies, including Burkitt lymphoma, follicular lymphoma, diffuse large B-cell lymphoma (DLBCL), mantle cell lymphoma, and multiple myeloma seem to have an overexpression of EZH2 and H3K27 methyltransferase enzymes as both inhibit genes responsible for suppressing tumor development [63]. Generally, HM have a mutation in UTX, which is the demethylase for H3K27 and its expression has been suppressed with use of epigenetic therapy especially in T-cell acute lymphoblastic leukemia [64,65]. However, after DNA DSB, both EZH2 protein and H3K27me3 epigenetic modifications are responsible in activating repair mechanism [66]. A study showed that the utilizing of histone deacetylase (HDAC) inhibitors alone or in combination with regular chemotherapies has multiple advantages: affecting DNA repair, reorganization of the converted H3K27me3-ac epigenetic switch and improved therapeutic efficacy in the EZH2 gain-of-function (GOF) mutant DLBCL cells [67].

Several pathways are involved in DNA repair, such as base excision repair (BER) or nucleotide excision repair (NER). BER is responsible for eliminating small, non-helix-distorting base aberrations in the genome; whereas, NER repairs and targets bulky helix-distorting damages, including repair of pyrimidine dimer which is caused by UV light [68]. Other genetic mutations such as insertion, deletion, and mis-incorporation of bases during DNA duplication and recombination are repaired by DNA mismatch repair (MMR). [69]. 

DNA is repaired by either non-homologous end joining (NHEJ) or homologous recombination repair (HRR) mechanisms activated after DNA DSBs. The NHEJ pathway does not use a complementary template and is mostly activated during the G1 phase or during the cell cycle [70]. In addition, it is induced not only as a response to DSB, but also in V(D)J recombination. Certain diseases are caused by mutation in NEHJ such as severe combined immunodeficiencies (SCID) and LIG4 syndrome [71]. HRR, on the other hand, functions in the late stage of the cell cycle (S and G2 phase). The stimulation of HRR is associated with the mutation of BRCA1/2 gene, which is directly related to hereditary breast and ovarian cancer [72]. 

Some tumors developed due to defective DNA repair mechanisms to keep the cell proliferating especially after the intervention of chemotherapeutic treatment. Thus, monitoring DNA repair activity seems to be essential in the patients under treatment to ensure that the therapy regimen in use has the desired effect on the DNA repair pathways with the aim of increasing the efficiency of drugs in cancerous cells [73]. 

## 6. Defects of Ataxia-Telangiectasia in Hematological Malignancies 

Ataxia telangiectasia (A-T) is an autosomal recessive disorder caused by genome instability or changes in the DNA damage response. A-T can affect the nervous, immune system, and other body organs whilst leaving the organs susceptible to cancer development. It affects different age groups, leading to immunoglobulin and antibody deficiencies as well as lymphopenia. The liability for cancer development, especially those of lymphoid origin, significantly increases in patients with A-T and the overall survival is estimated to be approximately 25%. In addition, various health conditions can accompany hematological cancers such as pulmonary diseases and dermatological disorders [74,75]. 

Mutations in the A-T (*ATM*) gene, which encodes the ATM protein is the main cause of A-T disease as the ATM protein is involved in organization of cellular signaling pathways in response to genotoxic stress that causes DNA DSB. Somatic ATM mutations are found in several types of tumors but are mostly present in hematological malignancies. Young patients around 20 years old with a classic A-T disease are predominantly diagnosed with lymphomas or leukemias. Whereas in adults, the incidence of both lymphoid tumors and solid tumors including breast, liver, gastric, and esophageal carcinomas is higher [76]. One study which evaluated cases of hematological cancer observed that inactivating mutations in both ATM alleles were seen in 45% of patients with T-cell prolymphocytic leukemia [77]. Furthermore, nearly 45% of mantle cell lymphoma cases had mutations which involved either truncated ATM protein or missense mutations disrupting the PI3K domain [78]. Most of the ATM gene in patients with B-cell chronic lymphocytic leukemia (CLL) is defective. This is observed in 20% of the cases due to loss of heterozygosity following deletion of chromosome 11q23. This mutation is characterized by shorter progression-free survival prior to chemotherapy and is associated with poor prognosis [79,80]. Next-generation sequencing has shown that ATM point mutations occur in at least 9% of CLL cases [81]. Classical Hodgkin lymphoma patients showed that the expression of ATM in Reed Sternberg cells is low. In addition, the amounts of ATM were not identified, unlike normal germinal center B cells, although the ATM promoter did not contain a loss of heterozygosity, pathogenic mutations, or hypermethylation. Therefore, changes in the upstream regulators are suspected to be involved in decreasing the expression of ATM [82].

It is difficult to monitor cancer development in individuals with the A-T disease, particularly for lymphomas and leukemias when compared with solid tumors. This is because there are various screening methods to diagnose and control disease progression such as mammography, colonoscopy, and PSA levels. Some specific symptoms such as swollen lymph nodes and fever of unknown origin are usually associated with the diagnosis of hematopoietic cancers [74].

## 7. Gene Mutations of DNA Damage Response in Hematological Malignancies

**Figure 1:**
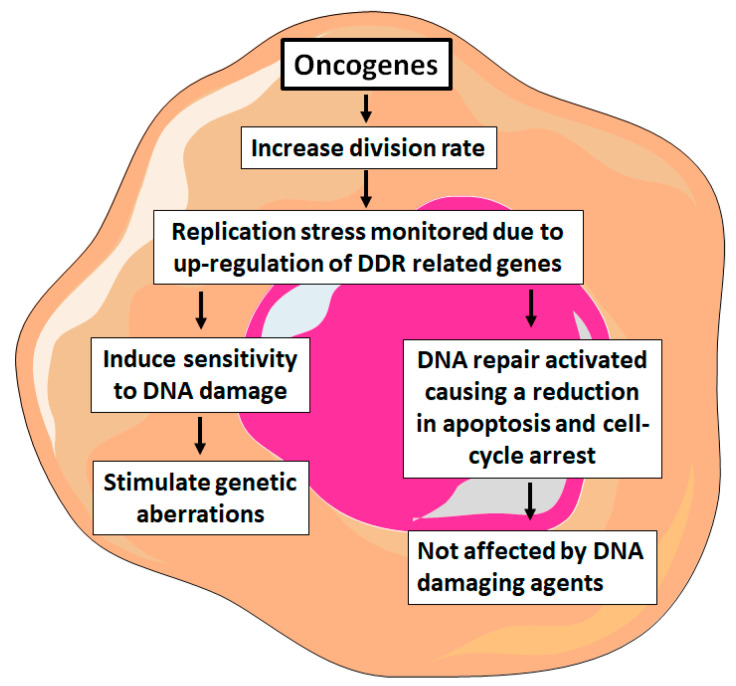
The DNA Damage Response (DDR) pathway in cancer cells. Several oncogenes are responsible for controlling cell division rate under cellular stress in leukemic cells.

Induction of DDR elements can occur as a response to replication stress caused by oncogenes during the rapid division of cancer cells [49,83]. This process may be responsible for tumor progression, particularly in early-stage solid tumors and with the formation of pre-neoplastic lesions. Several studies have shown that mutations in the MYC, BCR-ABL1, and FLT3 are associated with genes involved in DNA damage response [84]. Genomic instability is responsible for the progression of acute leukemia, caused by the dysfunction of the DDR genes and activation of certain oncogenes (Figure 1). Encouraging MYC-driven cells induce cell division rate and leads to monitor the expression of different genes that play a role in ATR/CHK1 and ATM/CHK2 pathways. However, the collapse of replication forks is mainly protected by highly activated ATR/CHK1 pathway in B-cell lymphomas as a result of MYC expression [85,86]. Besides lymphoma, the expression of MYC is also increased in patients with chronic myeloid leukemia (CML), translocations t(8;14), t(8;22) in ALL and in AML [87,88,89].

A recent study in leukemic cells reported that some activated kinases like BCR-ABL1 and FLT3/ITD arise from MYC-increased error-prone repair that lead to increased genomic aberrations [90]. The level of DNA damage was measured using γ-H2AX expression and activated CHK1m. A study revealed that both parameters are induced in AML patient samples with complicated karyotype compared with normal karyotype AML samples and normal hematopoietic precursors [91]. In addition, the expression of the kinase CHK1 in leukemic blasts of ALL patient samples is higher than in normal lymphoid precursors [92,93]. Furthermore, the ATR/CHK1 pathway decreases the effect of cytotoxicity of traditional treatments in order to regulate the level of generated DNA damage due to treatment, protect BCR-ABL1-positive leukemic cells, and reduce the progression of cell cycle phases. Together, these pathways allow the repairing mechanism to be more efficient in leukemic cells [94].

The ten-eleven translocation-2 (*TET2*) gene is located on chromosome 4q24. The expression of this gene was increased in myeloid malignancies, as the proteins of *TET2* family proteins are critically involved in DNA hydroxymethylation [95]. Mutated *TET2* leads to induction of 5-methylcytosine (5 mC), which is used as a biomarker in the diagnosis and prognosis of hematopoietic malignancies, particularly myeloid malignancies [96]. *TET* family members (1,2,3) affect either the DNA damage proteins or the repair response. Increase in DNA strand breaks was observed in the cells with reduced *TET1* expression [97]. During serious insults, such as DNA strand breaks, *TET1* plays a protective role in the cells by monitoring various essential DNA repair genes located in the promoter regions such as *RAD50*, *BRCA1*, *RAD51*, and *TP53BP1* [98]. A research study performed on a mouse model demonstrated that the main reason for DNA instability is the absence of *TET1* leading to the progression of myeloid malignancy [99,100]. In addition, *TET3* stimulates ATR-dependent DNA damage response and control DNA repair by facilitating the transformation of 5 mC to 5 hmC [101]. A study on cancerous cell lines elucidated that the sites of endogenous DNA damage are enriched with active 5-hydroxymethylcytosine (5 hmC) [102]. The chromosomal segregation disorder formed as a result of the absence of *TET2* due to DNA replication stress. These findings demonstrated that the expression of BRCA2 mRNA was reduced after *TET2* knockout [102]. The presence of both proteins BRCA1 and BRCA2 are essential, particularly in homologous recombination (HR) during the DNA damage response to maintain genomic stability. Other protection strategies against chromosomal instability is through improving the levels of 5-hmC which results due to increase of wild-type *TET2* [103]. Consequently, *TETS* has a crucial role in maintaining genomic stability through encouraging DNA damage repair pathway.

## 8. Treatment of Hematological Malignancies and its Effect on DNA Damage and Repair

**Figure 2:**
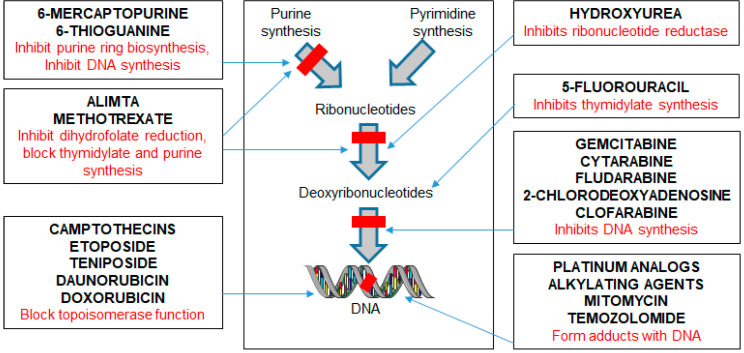
Classes of agents that are used in cancer therapy and their targets. Each treatment has a specific target in the cell proliferation starting from causing cell cycle arrest to disruption of DNA function. These chemotherapeutic agents can be used as single agents or in combination with a wide range of drugs for treating hematological cancers based on the type and stage of the hematological malignancy.

Various classes of agents currently in use for cancer therapeutics used in hematological malignancies are listed in Figure 2. They target either cell cycle, disrupt DNA function or proliferation of cancer cells. In the case of lymphoid malignancies, they are employed either as single agents or in combination according to the type and stage of the lymphoid malignancy [104]. Some of the standard therapies used in lymphoid cancers include cyclophosphamide (ifosfamide), vinca alkaloids (vincristine and vinblastine), anthracyclines (doxorubicin), bleomycin, bendamustine, gemcitabine, platinum analogues, and topoisomerase inhibitors (e.g., etoposide) [105]. Steroids such as prednisolone or dexamethasone are lympholytic and routinely combined with chemotherapy [106].

Immunotherapies such as immune checkpoint blockers (ICBs) have been used in the treatment strategy in various cancer types with many clinical trials showing that they improve treatment response and survival rate of individuals [107]. Nevertheless, few patients responded poorly to immunotherapy and therefore investigation of new biomarkers to identify responders from non-responders would help to understand the physiological differences between groups. Usually, the accumulation of mutations in cancer-related genes and the production of neoantigens result from disorder of DNA mismatch repair proteins and subsequent high microsatellite instability. These mutations promote the activation of anti-tumor immune response in the patient [108].

Recent studies demonstrated that cancer stem cells (CSCs) play a role in the activation of tumor-initiating cells and therapy resistance cells. Thus, the ability of anti-tumor treatment to enter the cancerous cells, which mainly consist of cancer progenitor cells (CPCs), is not enough to eliminate both CSCs and residual CPCs leading to the development of therapy refractoriness. Genotoxic stress, recombination-activating genes 1 and 2 (RAG1/2), activation-induced cytidine deaminase (AID), and cytotoxic treatment stimulate the survival of CSCs and/or CPCs due to altered DNA repair mechanisms. Therefore, targeting DNA repair pathways could significantly enhance the DNA damage in CSCs and CPCs. The lethal effectiveness of such treatment is seen in the cancerous cells addicted to DSB repair mechanisms [109].

## 9. ATM-Deficient Cancer Therapies

### 9.1. Poly ADP ribose Polymerase (PARP) Inhibitors

Several clinical trials have assessed PARP inhibitors alone or in combination with ATM inhibitor. Olaparib is a well-known PARP inhibitor that has been used in preclinical studies on patients with CLL, mantle cell lymphoma, and gastric cancer with ATM-deficient leukemic cells and demonstrated activity as a single-agent [12,110,111].

### 9.2. Targeting ATR

Treatments that target ATM loss of function and pathways mediating SSB DNA repair are promising avenues for therapy. A synthetic lethal siRNA screen applied on mantle cell lymphoma with deficient ATM showed an improvement in sensitivity to ATR inhibitors [112]. Preclinical data suggested that inhibiting this pathway may also be effective in other cancers. Several compounds have been used to inhibit ATR, including VE821, VE822, and AZD6738 [113,114]. Another study showed that ATR kinase inhibitor (AZD6738) has a significant effect on ATR particularly in cells with ATM dysfunction. A xenograft model revealed that the effect of anti-tumor drugs in ATM-deficient tumors such as ataxia telangiectasia mutated and rad3 related (ATR) kinase inhibitors was greater compared with the wild type. In addition, the DNA damage biomarker γ-H2AX was detected only in tumor tissues and was absent in normal bone marrow [115]. The maximum cytotoxicity effect was observed after treating the proliferating leukemic cells collected from CLL patients with AZD6738 [116]. This finding was also observed in CLL cells with mutated ATM or TP53 [117]. 

### 9.3. CHK1 Inhibitors

Several pre-clinical and clinical studies have been established to investigate the effectiveness of CHK1 inhibitors [118]. MK-8776, is another CHK1 inhibitor tested in clinical trials and is characterized by the prompt stimulation of γH2AX accumulation [119]. The ability of this inhibitor to produce DSBs was assessed as a single agent or in combination with various DNA damaging agents. This CHK1 inhibitor clearly manifested a significant increase in its efficacy when combined with other chemotherapies in phase II clinical trials [120]. This study indicates that MK-8776 treatment is more potent when combined with a histone deacetylase (HDAC) inhibitor (HDACI) in AML cell lines and AML primary cells [121]. The effectiveness of this therapeutic combination with MK-8776 inhibitor in AML cells does not rely on p53 mutation as the treatment mechanism depends on reducing the activity of CHK1, disruption of intra-S phase checkpoint, alteration, and reduction of DNA replication [121]. Moreover, the cytotoxicity of several nucleoside analogs (fludarabine, cytarabine, and gemcitabine) was improved in the presence of MK-8776 in the p53-mutated MEC1 cell line and primary CLL cells [122].

The efficiency of AZD7762 in immature KG1 AML cell line and primary cells from AML patients showed that the combination of AZD7762 (ATP competitive CHK1/CHK2 inhibitor) and melphalan (alkylating agents) induced cell death and reduced cell proliferation [123,124]. It also reduced DNA damage. In addition, the relation between the impact of checkpoint kinase inhibitors and a complicated karyotype is responsible for the poor response to standard therapy. Therefore, measuring γH2AX and CHK1 to track the basal level of DNA damage can be used to predict the optimal therapy of choice for AML patients [125]. 

PF-0477736, an ATP competitive CHK1 inhibitor, is used to treat several tumors with higher selectivity to CHK1 than CHK2. This inhibitor has also been assessed in leukemic patients. It has been reported that CHK1 expression is higher in T-cell acute lymphocytic leukemia (T-ALL) than normal thymocytes. While the viability of ALL primary and leukemia cell lines is reduced with PF-0477736, normal thymocyte cells are only slightly affected by the treatment in vitro [93]. PF-0477736 induces apoptosis by disrupting cell replication by inactivation of G2/M checkpoint [92].

Combination of PF-00477736 with bosutinib (SKI-606), a Src/ABL inhibitor, was investigated in BCR-ABL1-positive CML and ALL cells. This treatment strategy was performed in vitro and in vivo and showed a high response rate [126].

A novel CHK1/CHK2 inhibitor called LY2606368 stated to be an efficient checkpoint suppressor, causes increased induction of apoptosis after exposure to DNA damaging agent, leading to high levels of DNA damage, particularly in the cells at S phase of the cell cycle [127]. Recently, LY2606368 has been used in clinical practice after evaluating the impact of this inhibitor in the presence and absence of other compounds. This finding showed that LY2606368 highly sensitized both primary and leukemic cells to several agents such as antimetabolite, clofarabine, and to tyrosine kinase inhibitors (imatinib and dasatinib) [128]. In vitro study demonstrated that the effect of DNA damaging agents such as pemetrexed and cisplatin was improved in the presence of LY2603618 and this result was also confirmed in a tumor xenograft model. This combination was established in a phase I clinical trial in patients with late stage cancer progression to assess the potency of LY2603618 when combined with pemetrexed and cisplatin [129]. Another combination study performed in AML cell lines and primary cells (*n* = 26) between LY2603618 and BCL-2 inhibitor ABT-199 concluded that LY2606368 improves the effectiveness of ABT-199 by the increasing of cell death as a result of reduction in MCL-1 expression [130,131]. 

### 9.4. Nucleoside Analogues

Nucleoside analogues (NA), considered as essential types of antimetabolites, are used in the treatment of both hematological malignancies and solid tumors. The principle of mechanism of this therapeutic compound is close to the physiological nucleosides in terms of uptake and metabolism. As this compound is incorporated into recently synthesized DNA, it causes inhibition of its synthesis and chain termination [132].

Sapacitabine is a prodrug, orally administrated, and consists of a nucleoside analogue 2′-C-cyano-2′-deoxy-1-beta-D-arabino-pentofuranosylcytosine (CNDAC). It is used to induce cell apoptosis in ATM-deficient tumors. CNDAC integrates into the DNA and stimulates SSBs. When the cells enter the S phase, SSBs become DSBs. Homologous recombination is the repairing pathway that involves DSBs repair, but this pathway is absent in case of defected ATM [133]. A randomized phase II study performed on AML patients showed a 1-year survival rate in almost 35% of the selected group. Immune dysfunction is the most severe side effect of sapacitabine which includes cytopenias, febrile neutropenia, and infections [134]. Clinical trials on CLL patients are underway to evaluate the effect of sapacitabine in combination with cyclophosphamide and rituximab, particularly in all patients with del(11q) karyotype condition [135].

## 10. Conclusions

Pre-clinical and clinical trials are testing the use of cell-cycle checkpoint inhibitors, whether as a single agent or in combination with other drugs, to treat several cancers, particularly hematologic malignancies. Furthermore, most of the clinical trials discussed here show that the use of cell cycle checkpoint inhibitors in combination with chemotherapy or other agent has a major effect in improving the efficacy of the treatment. The response among different kinds of cancers, including acute leukemia subtypes was varied based on the sensitivity towards cell cycle checkpoint inhibitors [84]. Nevertheless, the proliferation of some clones might increase due to the reduction of DNA repair mechanism. The level of DDR and genetic instability in normal cells were measured in order to ensure the safety of using checkpoint inhibitors for a long period of time. 

The greatest challenge in sequential therapy that targets certain mutations or induces genetic instability in the treatment of various types of cancers is that it leads to proliferation of aggressive clones and hence development of therapy resistance. This cell behavior results in a significant reduction in the treatment response of cancerous cells towards traditional chemotherapeutic agents, leading ultimately to disease relapse. Therefore, use of checkpoint inhibitors has significantly improved the treatment response and survival rate through inducing genetic instability in cancer cells as a strategy to combat cancer. Furthermore, in this review, we have provided an overview of important kinases that regulate cell cycle checkpoints in order to inhibit the DNA repair pathway. Targeting such kinases provides a promising strategy to overcome resistance in the treatment of hematological malignancies.

## Abbreviations

AML:	Acute myeloid leukemia
ATM:	Ataxia telangiectasia mutated
BER:	Base excision repair
CIN:	Chromosomal instability
CLL:	Chronic lymphocytic leukemia
DDR:	DNA damage-response
DSB:	Double-strand breaks
GOF:	Gain-of-function
HDAC:	Histone deacetylase
HR:	Homologous recombination
MDS:	Myelodysplastic syndrome
MIN:	Microsatellite instability
MMR:	Mismatch repair
MMS:	Methyl-methane-sulfonate
NBS:	Nijmegen breakage syndrome
NCS:	Neocarzinostatin
NER:	Nucleotide excision repair
NHEJ:	Non-homologous end joining
ROS:	Reactive oxygen species
SDDC:	S phase DNA damage checkpoint
SSB:	Single-strand breaks
